# A comprehensive comparative study on microwave- assisted pyrolysis products derived from raw and digested organic waste, with emphasis on sewage sludge, food waste, and livestock manure

**DOI:** 10.1016/j.heliyon.2024.e29618

**Published:** 2024-04-16

**Authors:** Doo Young Oh, Daegi Kim, Ki Young Park

**Affiliations:** a Department of Civil and Environmental Engineering, Konkuk University, 120 Neungdong-ro, Gwangjin-gu, Seoul, 05029, Republic of Korea; b Department of Environmental Engineering, Mokpo National University, 1666, Yeongsan-ro, Cheonggye-myeon, Muan-gun, Jeollanam-do, 58554, Republic of Korea

**Keywords:** Organic wastes, Microwave-assisted pyrolysis, Waste to energy, R program, Correlation

## Abstract

This study focused on characterizing sewage sludge, food waste, and livestock manure, representative of continuously generated organic wastes, along with their anaerobic digestion residues. Microwave assisted pyrolysis was employed to investigate the relationship between the properties of the raw organic wastes and the resulting pyrolysis products, utilizing the R-program for analysis. Evaluation of the pyrolysis products of these six organic wastes revealed that char yield was primarily influenced by ash and fixed carbon contents, with higher yields observed in residues from anaerobic digestion compared to the original organic waste. Liquid and gaseous product quantities were found to increase with volatile content, while high-fat content within the volatile fraction notably enhanced liquid product yields, impacting syngas production. Analysis of syngas composition indicated a negative correlation between high nitrogen content in the feedstock and H_2_ generation. Furthermore, examining the correlation between chemical properties of organic waste and pyrolysis products revealed a proportional increase in protein components with nitrogen content, suggesting potential improvements in pyrolysis efficiency through raw material pretreatment enhancements by the R program.

## Highlights

• Assessment of microwave-assisted pyrolysis in various organic wastes.
• Comparison of the chemical characteristics of organic wastes to impact on pyrolysis was conducted.
• Assessing the impact of organic and biomass constituents on pyrolysis.
• Pyrolytic production was influenced by the content of fat and nitrogen.
• Using the R program, the relationship among the properties of pyrolysis products was scrutinized.".

## 1 Introduction

While the advancements in science and technology improve human life, they inevitably contribute to resource depletion and environmental pollution, emphasizing the critical need for sustainable development endeavors [1]. Amidst policy changes, the landscape of energy production methods is undergoing diversification, with a notable upsurge in enthusiasm for harnessing renewable energy sourced from organic waste [[2], [3], [4]].

The growing accumulation of organic waste, spanning from sludge and food waste to livestock manure [5,6], has been identified as a valuable energy or recycling asset; nevertheless, its optimal utilization has been impeded by policy and technical limitations Currently, the majority of organic waste undergoes recycling processes such as feeding, composting, and anaerobic digestion [[7], [8], [9]]. However, there is an urgent requirement for research into renewable energy methods to effectively harness the significant untapped energy potential of organic waste [10,11].

Anaerobic digestion has been consistently utilized for the treatment of municipal and industrial organic waste, offering the benefit of producing biogas as a renewable resource, along with generating electricity and heat [[12], [13], [14]]. However, anaerobic digestion produces a residue containing a high concentration of organic matter. This residue is called digested sludge and is classified as organic waste. Despite its high moisture content, digested sludge can be converted into fuel through thermochemical treatment, and research is being conducted to improve its energy recovery efficiency [15,16].

Among the thermochemical technologies, pyrolysis is an effective way to recover energy and resources from solid, liquid, and gaseous products [[17], [18], [19]]. According to the target product, pyrolysis can be classified into carbonization, liquefaction, or gasification. Pyrolysis can be classified as slow, fast, or flash, depending on the heating rate. In general, traditional pyrolysis mainly uses a method in which heat is transferred from the outside to the surface of the material through an electric or gas burner, which has the disadvantage of low heat transfer efficiency owing to the large size of the pyrolysis reactor. Microwave-assisted pyrolysis(MAP), which is characterized by the heat generated inside the material and transferred to the outside, was introduced to overcome this disadvantage. This makes heating more uniform and improves product quality [20]. In addition, microwave irradiation is not performed using an external heat source on the sample; therefore, rapid volume heating is possible [21]. The efficiency of converting electrical energy into heat is 80–85 %, enabling more efficient heating than conventional pyrolysis methods [22]. Heating through microwave irradiation has advantages over conventional heating methods, such as uniform and faster internal heating, ease of operation and maintenance, no need for feedstock grinding, and lower energy costs [23].

MAP of various feedstocks, including organic wastes such as sewage sludge [24,25], food waste [26], and livestock manure [27], has previously been studied. However, there is a lack of research on the characteristics of the pyrolysis products depending on the properties of the organic waste. Organic wastes have unstable physical and chemical properties, depending on their source, generation time, and treatment method [28]. A comparative analysis of the characteristics of organic wastes is necessary before they can be used as raw materials for MAP. In addition, it is necessary to analyze the properties of various organic wastes and correlate the mass balance and gas composition of the pyrolysis products with their properties.

Ongoing research on inorganic materials, which includes synthesis and modification efforts, is being complemented by characterization studies [29,30]. In addition to structural analysis of inorganic materials [[31], [32], [33]], there is potential for understanding their influence on microwave-assisted pyrolysis (MAP) of organic waste [34]. Although this investigation primarily tackled organic constituents, future studies are anticipated to incorporate information concerning inorganic materials, ensuring a comprehensive characterization of organic waste.

The aim of this study was to compare the gas products generated by microwave-assisted pyrolysis (MAP) using representative organic wastes—sewage sludge, food waste, and livestock manure—along with their residues post-anaerobic digestion. Characterization of the organic waste involved industrial, elemental, FT-IR, TGA, lignocellulose, and nutrient analyses, followed by a comprehensive analysis of material balance and product characteristics generated through MAP, facilitated by the R program, to establish correlations.

## 2 Materials and methods

### 2.1 Materials

The organic wastes used in the experiments were sewage sludge, food waste, and livestock manure, which were collected from anaerobic digestion facilities in South Korea. The samples used in the experiment were sewage sludge and its anaerobically digested residues, food waste and its anaerobically digested residues, livestock manure and its anaerobically digested residues, and six organic wastes. The samples were subjected to a thorough drying process in an oven maintained at 105 ± 5 °C for a period exceeding 24 h, followed by grinding into particles of <2 mm using a grinder before being introduced into the experimental framework. This method was applied to physicochemical analyses and MAP experiments.

### 2.2 Experimental methods

**Fig. 1:**
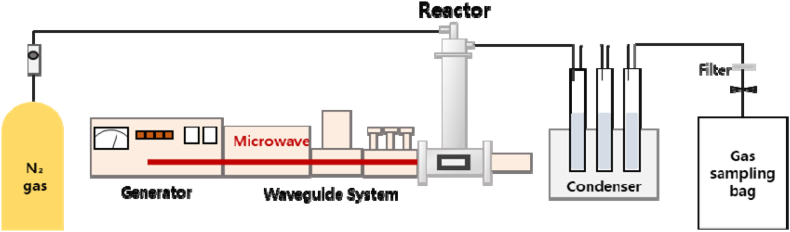
A schematic of the microwave-assisted pyrolysis reactor.

The MAP reactor used in the experiment was a microwave device manufactured by Korea High Frequency Co., Ltd., Korea, which can operate at a frequency of 2450 MHz and a maximum power of 1 kW. As shown in Fig. 1, the reactor consisted of a microwave generator, reactor, oil recovery device, and gas recovery device. The samples were injected into the reactor through a quartz tube, and the microwave power was controlled by measuring the temperature inside the reactor during the experiment.

The pyrolysis temperature was fixed at 900 °C based on the results of previous experiments [24]. Activated carbon was used as an absorber to increase and maintain the temperature inside the reactor via the microwave reaction. For the MAP experiment, the sample and absorber were mixed at a ratio of 8:2 (wt.%), and a total of 10 g was uniformly added. Previous research studies employed absorber quantities ranging from 7.5 % to 50 % to effectively elevate the target temperature of the microwave heat source [24,[35], [36], [37]]. This study was conducted with the goal of reaching the target temperature while minimizing the absorbent input, thus specifying the absorbent input ratio at 20 % (by weight) of the sample.After the sample was added, nitrogen gas flowed at 400 mL/min for 20 min to make the inside of the reactor anaerobic, and after the start of the reaction, it flowed at 100 mL/min to be used as a carrier gas for the product. After reaching the target temperature, the reaction time was maintained at 20 min, and all experiments were completed within 21–22 min. However, the rate of temperature increase differed according to the sample type. In the oil recovery device, the liquid product was recovered using dichloromethane, an organic solvent, and the gaseous product was recovered using a Tedlar bag for analysis. The solid product was recovered when the reactor was cooled to room temperature after completion of the reaction.

### 2.3 Chemical analysis

The recovery of pyrolysis products was determined by weighing the solid and liquid products. The weight of the gaseous product was calculated by subtracting the weights of the solid and liquid products from those of the input sample. The mass balance of the pyrolysis products was calculated from the amount of the recovered material. Elemental analysis was performed using a Flash 2000 elemental analyzer, in which trace amounts of sample (less than 1 g) were heated to high temperatures, complexed, and combusted in an oxygen environment. The gases produced were measured using a detector in the measurement system. The calorific value was analyzed using a Parr Model 1341 Plain Jacket Calorimeter, in which a 1 g sample was loaded into the calorimeter and burned in an oxygen bomb. The amount of heat generated was measured as the change in the temperature of the water surrounding the calorimeter.

The moisture content was determined by weight change after complete drying in an oven at 105 °C, and volatiles were measured based on additional weight loss by heating the coal to 950 °C in an N_2_ atmosphere. Ash was calculated as the weight of the residue remaining after combustion at 650 °C, and fixed carbon was calculated as the weight lost after combustion. Fourier Transform Infrared Spectroscopy (FT-IR) was performed using a Jasco FT/IR-4100 to measure the powdered samples through infrared irradiation and was calibrated with reference spectra to reduce the influence of the surrounding environment. Thermogravimetric analysis (TGA) was conducted using a TG 209 F3 instrument (Netzsch). For thermogravimetric analysis, nitrogen gas was used for anaerobic conditions, and the temperature was raised to 900 °C at a ramp rate of 5, 10, and 20 °C/min.

### 2.4 Organic matter content

Proteins in organic wastes were determined using a KjeltecTM8400 from Foss, based on the Kjeldahl method, in which 0.5–1 g of sample was digested with a digestion accelerator consisting of a mixture of potassium sulfate and copper sulfate (9:1 = W%:W%) and 10 ml of sulfuric acid, heated for up to 90 min, and titrated with a 0.1 N hydrochloric acid solution. Fat was analyzed using Ankom's A2000, XT 15, based on the ether extraction method, in which 2–3 g of the sample is dried for 2 h, poured with ether, and heated to 80 °C for 8 h to extract the fat, and the weight of the fat is obtained after recovering the ether. Ash and moisture were measured using Thermostable SOF–W155 and FHX-63 of Daehan Science Co. The carbohydrate content was calculated using the following equation (1).

(1) Carbohydrate(%)=100−Moisture(%)−Protein(%)−Fat(%)−Ash(%)

Lignin, cellulose, and hemicellulose contents were measured and calculated using an A2000 instrument from ANKOM Technology. The lignin content was determined by grinding the sample to within 1 mm, acidifying it with a 72 % H_2_SO_4_ solution, and calculating it before and after weighing. Cellulose and hemicellulose contents were calculated from the acid detergent fiber in the feed (ADF), neutral detergent fiber in the feed (NDF), and lignin content results. ADF measures the cellulose and lignin mix by digesting the sample with H_2_SO_4_ and Cetyl trimethylammonium bromide (CTAB; premixed chemical solution available from ANKOM) and analyzing the remaining residue. The NDF method is an analytical method that involves digesting a sample in a mild detergent solution and measuring the remaining residue, which can be used to identify cellulose, hemicellulose, and lignin mixtures. The neutral detergent is a chemical mixture of 30 g Sodium dodecyl sulfate, 6.81 g Sodium borate, 4.56 g Sodium phosphate dibasic dissolved in 1 L distilled water and 10 ml Triethylene glycol. The hemicellulose content was determined from the difference between the NDF and ADF values, and the cellulose content was determined from the difference between the ADF and lignin values.

### 2.5 Microwave-assisted pyrolysis(MAP) product analysis

The gaseous product, gas, was analyzed using a Gas Chromatography-Thermal Conductivity Detector (GC-TCD) from YL, and two types of columns (Supelco stock #13052-U and#13047-U) were used to measure the H_2_, CO, CO_2_, CH_4_, C_2_H_4_, and C_2_H_6_ gases. A 1 ml gas sample was injected through the inlet. The inlet temperature was 120 °C, the detector temperature was 150 °C, and the oven temperature was 50 °C for 5 min of 20 °C ramping, and the analysis was performed at 120 °C for 1 min after 3.5 min of ramping. Argon was used as the carrier gas.

### 2.6 Correlation analysis

Correlation analysis was performed using R studio, a free and open-source software, to analyze the experimental data of the organic waste characteristics and MAP products. The R program uses the Pearson correlation coefficient, which quantifies the linear correlation between two variables, X and Y, and is called Pearson's method. It is widely used in statistical analysis, pattern recognition, and image processing [24]. Equation (2) was used in the program R Studio to apply Pearson's method [38].

$$\bar{X}=\frac{1}{N}\sum_{i=1}^{N}X_{i}$$

$$\bar{Y}=\frac{1}{N}\sum_{i=1}^{N}Y_{i}$$

(2) $$r_{XY}=\frac{\sum_{i}^{N}\left(X_{i}-\bar{X}\right)\left(Y_{i}-\bar{Y}\right)}{\sqrt{\sum_{i}^{N}{\left(X_{i}-\bar{X}\right)}^{2}}\sqrt{\sum_{i}^{N}{\left(Y_{i}-\bar{Y}\right)}^{2}}}$$

Where rXY is Pearson's product-moment correlation and X and Y are variables in the dataset (X1, Y1), …,(XN, YN) [25].

The value of r was between −1 and 1. When r = 0, no linear relationship exists between variables X and Y. If r is positive, the value of one variable increases and the value of the other variable also increases; if r is negative, the value of one variable increases, and the value of the other variable decreases. An r^2^ value of 0.9 indicates that 90 % of the variation in the value of variable X is explained by the variation in the value of variable Y, and the variation in the value of variable X explains 90 % of the variation in the value of variable Y.

## 3 Results and discussion

### 3.1 Chemical properties of organic waste

The moisture content of the organic waste was mostly high (above 90 %), except for the anaerobic digestion residue of livestock manure, which had a slightly lower value of 75.26 % because it was collected after mechanical dewatering. The volatile content was the lowest for the food waste anaerobic digestion residue at 47.41 %, which is thought to be a result of the high biodegradability of food waste, as shown in Table 1 [39]. This was also confirmed by the nutrient and lignocellulose analyses shown in Table 1, where the raw food waste had a higher content of carbohydrates, proteins, and fats than the other organic wastes (83.95 %). Raw food waste had the lowest lignocellulosic content (9.82 %), including 3.44 % lignin, which is relatively difficult to biodegrade.

**Table 1:** The chemical and lignocellulosic properties of organic wastes (wt.%).

	Raw organic waste			Digested organic waste		
	Sewage sludge	Food waste	Livestock manure	Sewage sludge	Food waste	Livestock manure
Moisture	94.37	89.86	90.29	98.39	97.01	75.26
Volatile matter^a^	67.53	79.49	70.09	63.33	47.41	69.81
Fixed carbon^a^	6.91	9.24	7.94	5.47	7.60	18.05
Ash^a^	25.56	11.27	21.96	31.20	44.99	12.15
Heating value^a^ (kcal/kg)	4242.46	5019.82	4630.02	3448.31	2813.48	4381.07
Carbon^a^	38.72	47.91	42.62	32.64	28.53	43.21
Hydrogen^a^	6.00	7.09	6.21	5.03	3.83	5.36
Oxygen^a^	23.06	29.23	24.43	25.37	18.17	34.87
Nitrogen^a^	5.72	4.22	3.85	4.64	3.34	3.08
Sulphur^a^	0.94	0.29	0.93	1.13	1.14	1.33
Carbohydrate^a^	28.78	40.38	36.64	31.02	26.17	64.65
Protein^a^	36.79	28.25	25.36	29.63	22.02	20.23
Fat^a^	7.30	19.65	16.25	2.99	3.14	0.39
Other^a^	27.14	11.73	21.75	36.36	48.66	14.72
Lignin^a^	10.62	3.44	5.03	16.15	8.28	32.48
Cellulose^a^	2.76	2.73	5.81	1.83	1.07	9.90
Hemicellulose^a^	7.50	3.65	11.11	9.85	8.49	13.03
Other^a^	79.12	90.18	78.05	72.17	82.16	44.59

a dry basis.

Calorific value analysis showed a calorific value of more than 3400 kcal/kg for all samples except the food waste anaerobic digestion residue. The low calorific value of food waste anaerobic digestion residue was due to its high ash content (44.99 %). The lignocellulose content in all organic wastes was very low (less than 28 %), except for the lignocellulose percentage of 55.41 % in the livestock manure anaerobic digestion residue. As a result, the lignocellulose content is expected to significantly impact the pyrolysis of organic waste. Therefore, the nature of the organic waste may be an important factor influencing its pyrolysis reaction mechanism of organic waste. In addition, nutrients such as carbohydrates, proteins, and fats vary in content from 52 % to 88 % in organic waste, which can be an important factor in estimating the composition of pyrolysis products.

**Fig. 2:**
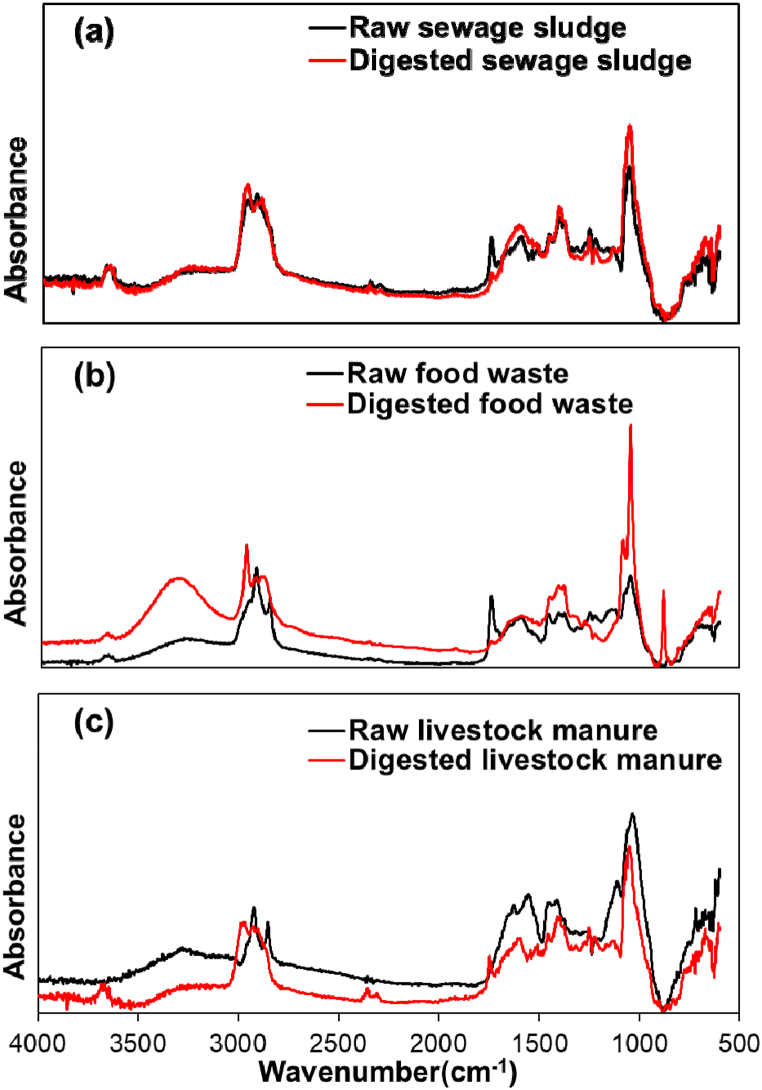
Changing the chemical structure of organic wastes through FT-IR; (a) sewage sludge, (b) food wastes, and (c) livestock manure.

The chemical structure of the organic wastes was examined by FTIR analysis, and the changes in the chemical structure of the organic wastes through anaerobic digestion are shown in Fig. 2. The distinct peak difference between the sewage sludge and the sewage sludge anaerobically digested residue is the decrease in the C=O stretching peak at 1720 cm^−1^, which indicates the decomposition of ketones, aldehydes, and carboxylic acids. The anaerobic digestion residue shows an increase in the C–O stretching peak at 1083 cm^−1^, confirming decomposition. This indicates the formation of abundant O-containing groups as C–O stretching peaks of carbohydrate and alcohol functions by biological degradation [40]. The changes in the FT-IR spectra of food waste, digested food waste, livestock manure, and digested livestock manure varied. Compared with the raw food waste, the O–H stretching peaks between 3000 and 3500 cm^−1^ increased in the digested food waste. This is believed to be due to the hydrolysis of the carbohydrate component of the food waste, which results in the degradation of polysaccharides to monosaccharides, indicating the presence of multiple OH groups in the monosaccharides.

The 900 cm^−1^ peak observed in the digested food waste had a C–H aromatic structure, indicating that the aromatic proteins in the food waste were broken down into amino acids. In addition, food waste showed stronger C–O stretching peaks for carbohydrate and alcohol functional groups than sewage sludge. In livestock manure, the decrease in the 1600 cm^−1^ peak confirmed the degradation of the C=C stretching of aromatic rings through anaerobic digestion. In contrast to food waste, the 3000–3500 cm^−1^ peak was reduced in digested livestock manure. This is contrary to the results of the anaerobic digestion of food waste, suggesting a higher content of polysaccharides than monosaccharides in digested livestock manure. The peaks between 2800 and 3000 cm^−1^ can be explained by the lignocellulose content, along with the C–H stretching peaks [41]. As shown in Table 1, the lignocellulose content was higher in the digested livestock manure than in the original livestock manure.

**Fig. 3:**
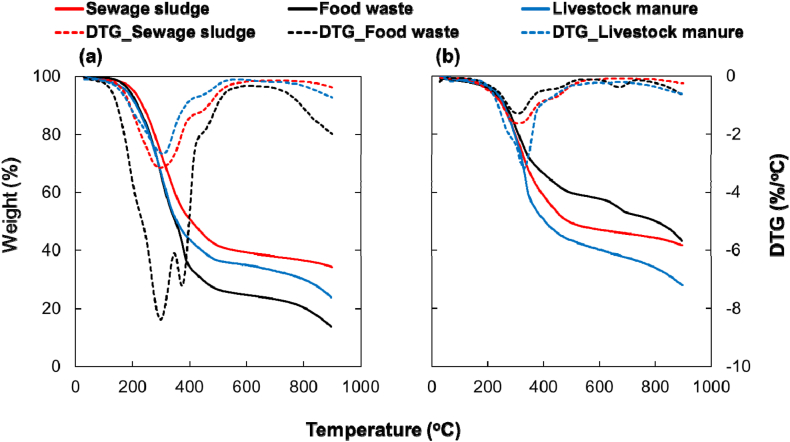
Thermogravimetric analysis of organic waste: (a) raw organic waste and (b) digested organic waste.

As shown in Fig. 3, the organic waste types were analyzed using TGA and the pyrolysis characteristics of the anaerobic digestion process. All organic wastes exhibited a weight loss of more than 56 %. The lowest weight loss was 56.8 % for food waste anaerobic residue, and the highest weight loss was 86.3 % for food waste. For all three types of organic waste, the anaerobic residues showed lower weight loss than the raw material. An inverse trend of lower weight loss was observed with higher ash content, as determined by the proximate analysis. The digested livestock manure had an ash content of 12.15 %, which was lower than that of the raw livestock manure (21.96 %); however, the weight loss was lower. This is likely due to the difference in fixed carbon content, with digested livestock manure having 18.05 % fixed carbon and raw livestock manure having 7.94 % fixed carbon. More weight loss can be achieved if pyrolysis methods that promote secondary decomposition of char are applied. The fat content of food waste was the highest (19.65 %), followed by that of livestock manure (16.25 %). The weight loss rate at 200–500 °C pyrolysis temperature was also higher in the order of food waste and livestock manure, which is expected to generate more liquid products.

### 3.2 Microwave pyrolysis

**Fig. 4:**
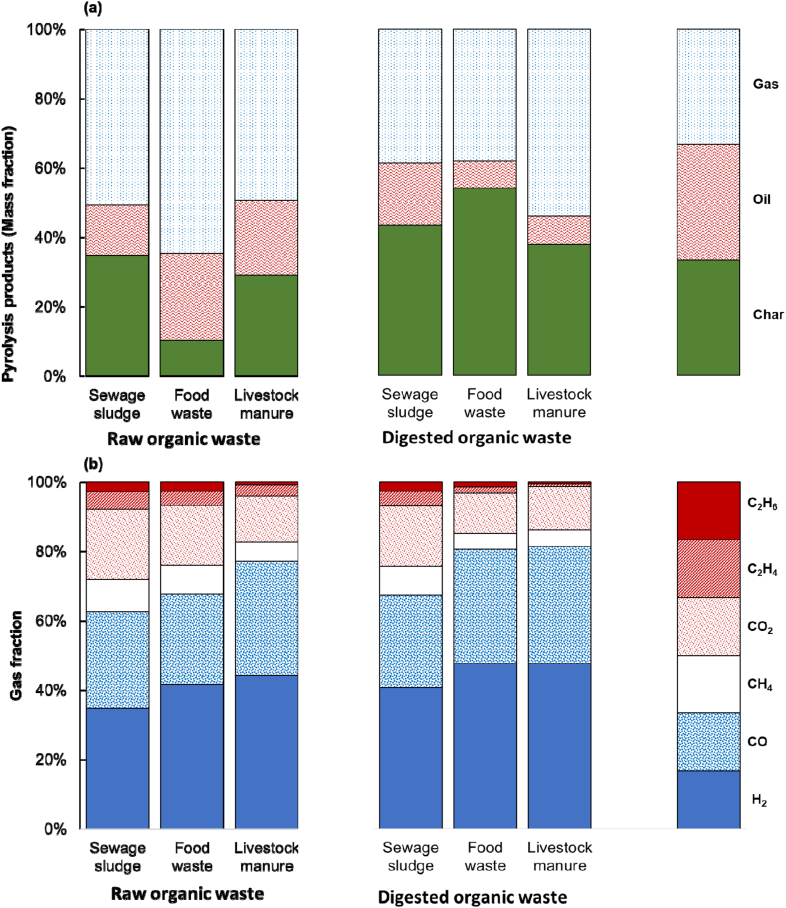
Microwave-assisted pyrolysis results: (a) mass balance of products and (b) syngas components.

The results of the MAP showed the mass balance of the products, as shown in Fig. 4 (a). The amount of char, a solid product, varies from 10 to 54 %, with the highest amount originating from digested food waste. In contrast to the thermogravimetric analysis results, the char yield of the digested sewage sludge was 43.27 %, which was higher than that of the digested livestock manure (37.72 %). This was due to the secondary decomposition of char at a high temperature of 900 °C, which resulted in greater decomposition of the digested livestock manure, which had a higher fixed carbon content. For liquid products, as expected from the results in Section 3.1, food waste, and livestock manure, pyrolysis products contributed the most at 25.01 % and 21.55 %, respectively.

Gaseous products were generated from raw food waste (64.66 %), digested livestock manure (54.05 %), and raw sewage sludge (50.75 %). Raw food waste showed high oil production but also high gas production, which is likely a result of its high volatile content due to its high-fat content. Raw livestock manure, which had the second highest volatile content, had a smaller gas fraction than digested livestock manure and raw sewage sludge because of the higher amount of liquid products. However, the gas fraction of the raw livestock manure was insignificant (49.34 %).

Fig. 4 (b) shows the composition of the syngas produced by MAP. The H_2_ content of the syngas generated by the MAP of digested organic waste was higher than that of raw organic waste. Sewage sludge showed a 17.21 % increase in H_2_ generation from the residue after anaerobic digestion. The smallest difference between raw and digested waste was observed for livestock manure, with a 7.59 % increase. An increase of 14.41 % was observed for the digested food waste. The H_2_/CO ratio of the gas produced from raw organic waste was 1.25–1.59, and the H_2_/CO ratio of the syngas produced from digested organic waste was 1.41–1.53. In contrast to the increase in H_2_ production, decreases in C_n_H_m_ and CO_2_ production were observed in the syngas generated from the digested organic waste. The increases and decreases in CO production from the digested organic waste were insignificant. This suggests that the nature of the organic waste changed during anaerobic digestion and that thermochemical treatment helped degrade polymeric materials into small molecules more easily.

### 3.3 Correlation between the properties of organic waste and microwave-assisted pyrolysis products

**Fig. 5:**
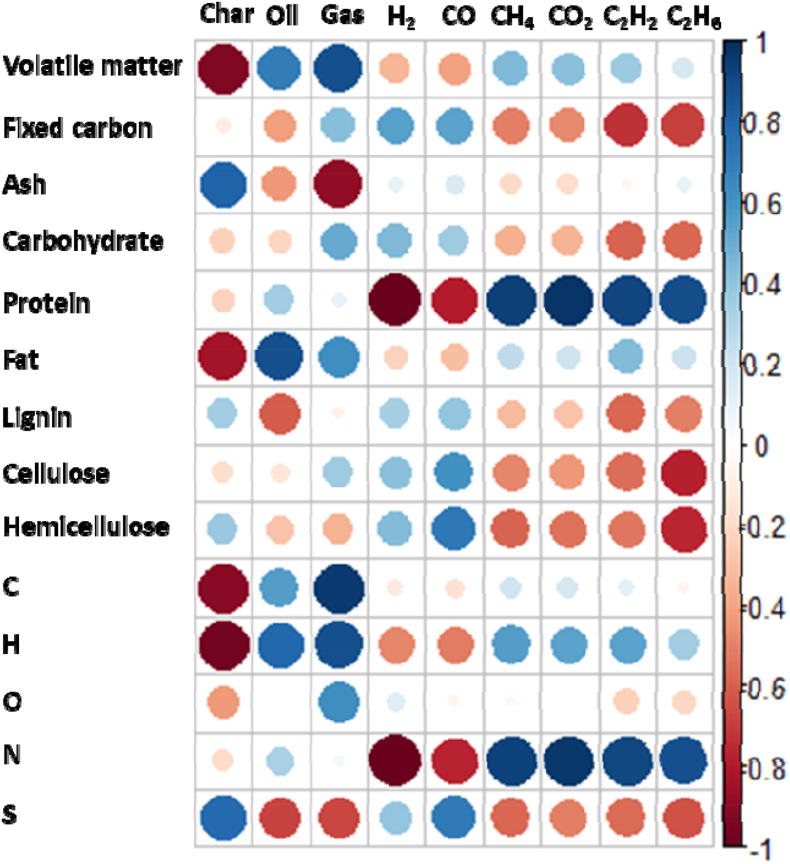
Correlation between the properties of organic wastes and pyrolysis products.

The chemical properties of the organic waste analyzed earlier, biomass composition analysis, and efficiency of the pyrolysis products were compared using the R program. The results were compared in various ways to correlate the pyrolysis efficiency with the characteristics of the organic material, such as the efficiency of pyrolysis products, biomass composition, and elemental analysis. Fig. 5 shows the correlation between the effects of organic waste characteristics on the MAP results. The characteristics of the organic waste were determined using industrial, nutrient, lignocellulose (lignin, cellulose, and hemicellulose), and elemental analyses. The pyrolysis results were based on the mass balance between the pyrolysis products and the syngas composition.

The higher the content of volatile and fixed carbon in the organic waste, the higher is the gas generation. However, the compositional analysis of the generated syngas showed conflicting results for volatile and fixed carbon. The higher the volatile fraction, the lower the H_2_ and CO contents, and the higher the fixed carbon content, the higher the H_2_ and CO contents. In addition, the higher the volatile fraction, the higher the oil and gas production. This result shows that the quality of the feedstock input for MAP is more suitable for pyrolysis gas generation when the quality of the feedstock is constant and is also an important factor affecting the production of constant-quality char and oil. The correlation also showed that feedstock quality affected syngas composition. This was evidenced by the increased H_2_ and CO content ratios of syngas from pyrolysis gas generation using anaerobic digestion residues, the quality of which was more consistent with that of biological treatment.

The correlation of the pyrolysis gas with the content of lignocellulosic components such as lignin, cellulose, and hemicellulose showed that a higher lignocellulosic content had a positive effect on increasing H_2_ and CO contents. Among the three components, lignin and hemicellulose were more closely related to the increase in char content than to the gas and oil content. The cellulose component was found to have a negative effect on char and oil production and a positive effect on gas production.

**Fig. 6:**
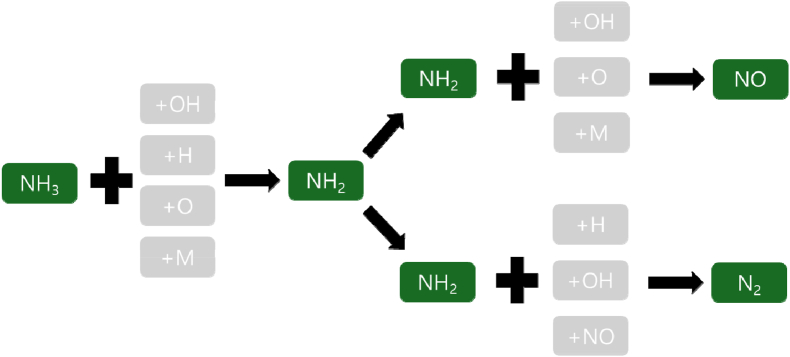
The behavior of nitrogen in the products of pyrolysis.

The correlation between the nutrient analysis of the organic waste and MAP results showed that carbohydrates were the most favorable components for gas production during pyrolysis (Fig. 5). Gas composition analysis showed that carbohydrates positively affected the increase in H_2_ and CO contents, whereas protein and fat components negatively affected the increase in H_2_ and CO contents. Proteins and fats also had some effect on gas production, but they had a greater effect on oil production than on gas production. In particular, proteins showed a very negative trend in H_2_ and CO content, which are the end products of MAP. This was confirmed by correlation with the elemental analysis results. The N component of the organic waste was mostly contained in proteins, and the correlation graphs of N and proteins were consistent. It is believed that controlling the N component is essential for producing high-quality syngas through the MAP of organic waste. According to previous studies, the majority of nitrogen present in biomass manifests as NH_3_ and N_2_, with only minute traces detected as HCN and NO [42]. Biomass characterized by a high nitrogen content can serve as a sustainable source of both nitrogen and hydrogen [43]. As depicted in Fig. 6, the nitrogen component of the raw material is predominantly released as NH_3_, N_2_, NO, etc., with NH_3_ constituting the major portion. Consequently, it is hypothesized that the production of NH_3_ will influence the quantity of H_2_ generated. However, a more precise understanding of this mechanism necessitates further investigation.An interesting finding from elemental analysis is that O reduces char production and helps increase gas production. This is the result of secondary decomposition owing to the oxidation of char and oil by the O component of the element.

## 4 Conclusion

Organic waste has many properties that make it difficult to use as a feedstock for MAP processes. In this study, the correlation between the properties of the organic waste and pyrolysis products was examined. The higher the fixed carbon content in the organic waste, the more significant the gas yield and H_2_ and CO contents, especially char generation. Higher volatile matter content increased gas and oil yields but decreased the H_2_ and CO contents. Cellulose content had a negative effect on char and oil yields but a positive effect on gas yields, whereas lignin and hemicellulose were strongly associated with increased char yields. The H_2_ and CO contents, which are thought to be indicative of gas properties, were positively influenced by carbohydrates and negatively influenced by proteins and fats. Because the protein content of organic waste is proportional to the nitrogen content of the product, it is assumed that organic waste with high protein content would be less valuable as a pyrolysis feedstock. The digested organic waste was stabilized by biological treatment, resulting in a decrease in fat and protein contents and an increase in H_2_ and CO. Organic wastes characterized by high fixed carbon and O contents and low protein and N contents appear to improve the quality of the pyrolysis products.

## CRediT authorship contribution statement

**Doo Young Oh:** Writing – original draft, Validation, Resources, Formal analysis, Data curation. **Daegi Kim:** Writing – review & editing, Writing – original draft, Project administration, Formal analysis, Conceptualization. **Ki Young Park:** Writing – review & editing, Writing – original draft, Project administration, Formal analysis, Conceptualization.

## Declaration of competing interest

The authors declare that they have no known competing financial interests or personal relationships that could have appeared to influence the work reported in this paper.
